# Physical Properties of Nanoparticles That Result in Improved Cancer Targeting

**DOI:** 10.1155/2020/5194780

**Published:** 2020-07-13

**Authors:** Randa Zein, Wissam Sharrouf, Kim Selting

**Affiliations:** ^1^University of Genoa, Department of Oral Diagnosis, Genoa, Italy; ^2^Lebanese University, Department of Oral Diagnosis, Beirut, Lebanon; ^3^Department of Veterinary Clinical Medicine, University of Illinois, Champaign-Urbana, IL, USA

## Abstract

The therapeutic efficacy of drugs is dependent upon the ability of a drug to reach its target, and drug penetration into tumors is limited by abnormal vasculature and high interstitial pressure. Chemotherapy is the most common systemic treatment for cancer but can cause undesirable adverse effects, including toxicity to the bone marrow and gastrointestinal system. Therefore, nanotechnology-based drug delivery systems have been developed to reduce the adverse effects of traditional chemotherapy by enhancing the penetration and selective drug retention in tumor tissues. A thorough knowledge of the physical properties (e.g., size, surface charge, shape, and mechanical strength) and chemical attributes of nanoparticles is crucial to facilitate the application of nanotechnology to biomedical applications. This review provides a summary of how the attributes of nanoparticles can be exploited to improve therapeutic efficacy. An ideal nanoparticle is proposed at the end of this review in order to guide future development of nanoparticles for improved drug targeting in vivo.

## 1. Introduction

The first discovery of enhanced permeability and retention (EPR) by Matsumura and Maeda [1] and coworkers, demonstrating the potential for accumulation of macromolecules by extravasation through fenestrated blood vessels in tumors, has opened the door for many attempts to produce a drug able to reach the tumor site [1]. A century has passed since this discovery and yet only 1 in 10 drugs gain Food and Drug Administration (FDA) approval, primarily due to the lack of efficacy in later stage clinical trials [2]. FDA-approved nanomedicines for cancer therapy include doxorubicin (Doxil/Caelyx) [1], daunorubicin (DaunoXome) [2] and paclitaxel (Abraxane) [3], all of which show a modest improvement in the overall survival of patients [3, 4].

Tumor vasculature is well characterized as hyperpermeable, immature, and with elevated interstitial fluid pressure, all of which are conducive to an EPR effect. This effect can vary significantly, not only among patients, but also across different tumor types and even changes for the same tumor over time [5].

**Figure 1:**
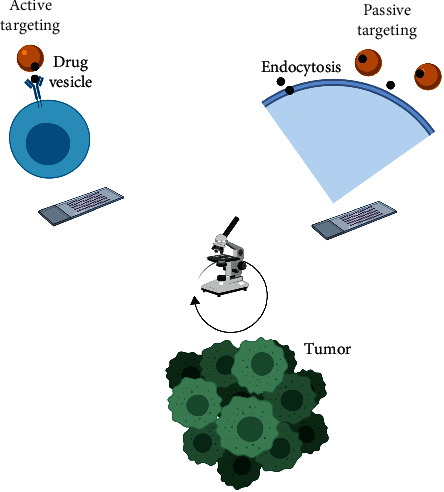
Active versus passive tumor targeting. In active targeting, the drug needs a receptor at the tumor surface, whereas in passive targeting, the drug enters the target cells passively.

An ideally designed NP should avoid clearance by the mononuclear phagocytic system, should remain in the blood circulation for a long time to ensure sufficient accumulation in the targeted tissues, should be internalized by the target tissue, and finally should have low toxicity. Modifying the physical properties of NPs such as size, charge, and shape, could result in changes in the therapeutic efficacy [6]. Two strategies for drug targeting are widely used: passive and active targeting (see Figure 1). Passive targeting is based on drug accumulation in tumor tissue due to the physical characteristics of both the drug carrier and the tumor architecture [7]. In contrast, active targeting is based on molecular ligand-receptor interactions and is only possible when the receptor and ligand come in close proximity (less than 0.5 mm) after the drug has circulated through the blood and extravasated in the tumor tissue [7]. In vitro interactions of NP with cells might not correspond to their behavior in vivo [8]. Therefore, by gaining deeper insight into interactions of NPs with cells and the tumor microenvironment, we may begin to maximize the potential of nanomedicine in cancer. This review will address physicochemical parameters affecting biodistribution and those affecting tumor uptake in order to propose characteristics of an ideal NP.

## 2. Nanoparticles First Interaction in Body: Protein Corona and Biological Barriers

Once introduced in the human body, NPs will face many obstacles before eventually interacting with the tumor including the protein corona and other biological barriers.

### 2.1. Protein Corona and NPs

**Figure 2:**
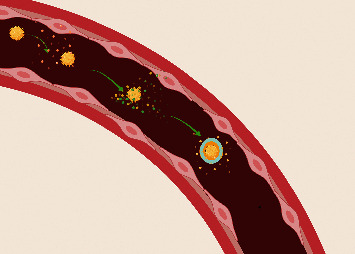
This figure shows how NPs are coated with proteins once they enter the blood circulation; after a short period of time, proteins came in close contact and form soft corona, and then a final hard protein corona is formed around the NPs containing a fingerprint specific for each individual and tumor.

Nanoparticles are being intensely researched as vehicles to deliver therapeutic drugs to a diseased site. It has become clear that slight changes in the physicochemical properties of NPs have significant biological implications. Most NPs that come into contact with biological materials are coated by a wide variety of proteins, which is named the “protein corona.” One component of the NP corona (called opsonins) can enhance the NP uptake by the RES. Under physiologic conditions, the corona may alter the NP properties by masking its surface characteristics [9, 10]. The exposure time in the blood circulation has been recognized as a key factor that shapes the NP biomolecular corona; furthermore, the new properties that are imparted to the NPs by the corona are the main factor that controls the distribution, nanotoxicity, and the therapeutic effect of NPs in the body [11] (Figure 2).

Some studies have suggested that the formation of the protein corona is an undesirable process; however, others have discussed the advantages of this formation, such as reducing the cytotoxic effect, eliminating undesirable interactions with the immune system, and facilitating cellular internalization [12]. The protein corona might reduce the targeting capability of the NPs by shielding the recognition site of the targeting ligands from the receptors [13]. Different disease types can have different effects on the corona composition, because for every disease, there are different types of proteins secreted in the body, and these proteins may affect the corona composition around the NPs [14]. These considerations mean that patients with different types of cancer may have specific types of coronas [14]. This has led some authors to introduce a new concept for NP targeting called a “personalized protein corona” (PPC) [15], wherein precise information on corona composition is a must to produce optimum personalized nanomedicines for therapy and diagnosis of every type of cancer. Colapicchioni et al. [16] investigated whether the pathological alteration of plasma proteins influenced the protein corona. They incubated liposomes with the plasma taken from patients with confirmed breast, pancreatic, and gastric cancer. The NPs were then isolated by centrifugation, and they found that the hard corona varied between patients having the same disease. They concluded that individual or personalized NPs can reduce the undesirable side effect of medications. NPs in the blood can function as scaffolds that attract specific proteins from the plasma; this may be due to the fact that tumor antibodies appear early in the plasma of patients and these antibodies are specific for each patient and disease [16].

As was said before, a single change in the physical properties of NPs may change the composition of the protein corona. Treuel and Nienhaus [17] found that the composition of the corona was affected by the size of NPs. Proteins tend to accumulate more on larger-sized NPs (100 nm) than on smaller-sized NPs, whereas proteins with higher affinity tend to bind more to smaller NPs (20 nm) [17]. The nature of the protein corona could influence the NP-cellular interactions (internalization) since the corona is the first point of contact between the NPs and the tumor cells [17].

For example, immunoglobulin binding to the NPs is called particle opsonisation and as a consequence leads to a rapid receptor-mediated phagocytosis uptake [18]. Furthermore, a higher degree of protein coverage has been found on hydrophobic NPs, compared to similar-sized hydrophilic NPs [19].

The corona may also increase the targeting capability of the NPs, if the binding site of the NPs is governed by the protein corona itself. In order to exploit this mechanism, it is necessary to understand which proteins deliver the NPs to which location [20]. As one example, Aoyama et al. found that the cluster in the protein corona could play a key role in the stealth effect by inhibiting the cellular uptake of silver NPs by phagocytic cells (macrophages) [21]. Furthermore, the plasma-protein corona suppressed the macrophage uptake more efficiently than the serum-protein corona due to the higher amount of cluster plasma [21].

At the present time, a large gap still exists in the understanding of the basic laws that govern the protein corona formation. Since the corona is the first interaction between the NPs and the tumor, it is essential in the future to establish a mathematical model to predict NP-surface interactions. What is clear now is that the final corona around the NP contains a so-called “fingerprint,” which is related to the type of tumor, the physical characteristics of the NPs, and the stage of the tumor.

### 2.2. NPs and Biological Barriers

Once administered, NPs may encounter many obstacles in reaching the target site. For intravenously administered NPs, the first barrier is the reticuloendothelial system (RES) consisting of the liver and spleen, which rapidly removes many particles from the circulation. In addition, the endothelium of blood vessels within the target tissues is also a barrier [22]. In healthier tissue, NPs cannot cross the endothelium of the blood capillaries, whereas in some pathological conditions, for example, inflammation or cancer, the cells of the endothelium lose the integrity of their connections due to the influence of proinflammatory cytokines. The gap between the endothelial cells is increased so that the NPs can extravasate from the vessel system into the diseased tissue. Therefore, the leakiness in tumor vasculature leads to better penetration of the NPs and then retention of the NPs in the tumor bed in a process known as “enhanced permeability and retention” [23].

If the NPs succeed in escaping from the blood capillaries, they face a third barrier in the interstitial space composed of collagen and elastic fibers composed of glycosaminoglycans and other proteins that form the extracellular matrix (ECM). In diseases such as liver fibrosis and neoplasia, the collagen content is higher than that of normal tissues [24]. Because of this, the excessive rigidity and increased interstitial pressure of the ECM pose a barrier to NP transport from the capillaries to the target cells [24, 25].

Some NPs will release their contents spontaneously once they have extravasated into the tumor, while other release their contents in response to a stimulus such as hyperthermia, laser exposure, or magnetic fields. The released drug will interact as usual with nearby cells. Because NPs cannot simply enter the target cells via diffusion, the next barrier for NPs is the plasma membrane. The mechanisms by which NPs are internalized by the target cells include pinocytosis, phagocytosis, or endocytosis [3].

The mechanism of internalization depends on the NP properties as well as the size and type of cells involved. If NPs are released from endosomes and lysosomes, they can diffuse in the cytoplasm and could enter the cell nucleus. Usually the membrane of the nucleus does not allow entry of NPs larger than 9 nm, providing yet another barrier [26].

## 3. Physicochemical Properties of Nanoparticles Affecting Biodistribution

The two most commonly studied parameters that affect NPs biodistribution are the size and the shape of nanoparticles; however, the charge and the coating surface of the NPs may play some role in biodistribution.

### 3.1. Effect of NP Size on Tissue Biodistribution

The therapeutic effect of NPs can be limited by their nonspecific systemic biodistribution, which can cause systemic toxicity and lead to reduced concentrations of drug delivered into the tumor (less than 5%). In order to enable diverse application of NPs, it is crucial to study the biodistribution of different-sized NPs, to gain a clear idea of what size NPs to use and for what kind of treatment.

Sonavane et al. [27] evaluated the biodistribution of AuNPs with different size (15, 50, 100, and 200 nm) after intravenous administration and found that the accumulation of NPs in various tissues was size-dependent; the smallest NPs (15 nm) showed the highest accumulation in organs (liver, lung, spleen, and kidney). Only the smallest NPs (15 nm) were able to cross the blood brain barrier [27, 28].

The size of the NPs will also affect their clearance from the circulation. Renal clearance is very rapid for particles with diameters smaller than 5-6 nm, while clearance by the liver and the spleen is rapid for larger particles, above 200 nm in diameter [29]. Particles with sizes 200 nm or larger are mostly removed by the mononuclear phagocytic system (MPS, also known as the RES), mediated by cells in the liver, spleen, and bone marrow [30]. At 100 nm, NPs have poor diffusion within the dense collagen matrix of the interstitial space, thus resulting in poor penetration into the tumor parenchyma and restricted NP accumulation around tumor blood vessels [30].

**Figure 3:**
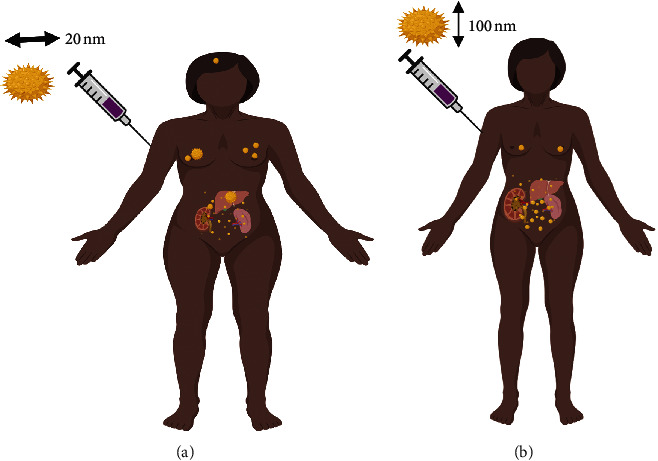
Effect of varying the NPs size on tissue biodistribution of patient with breast cancer. A 100 nm NPs mostly distribute in the liver, spleen, and kidney, and traces could be found in the breast, whereas, for 20 nm NPs, they mostly distribute in the kidney, spleen, and liver; a moderate amount is able to reach the breast tumor and traces were found in the brain.

Cytotoxicity is also affected by NP size; the smaller the size, the greater the toxicity. Gao et al. [31] measured the cytotoxicity of different NPs with sizes ranging from 8 nm to 37 nm and found that the 8 nm NPs showed more cytotoxicity compared to the larger size NPs [31]. In Table 1, we summarize how variation in the size NPs affects their biodistribution (Figure 3).

### 3.2. Effect of NPs Shape on Biodistribution

While NP size is the principal parameter that affects macrophage uptake, the shape of the NP also plays a major role in enhancing or inhibiting the uptake and biodistribution [36].

**Figure 4:**
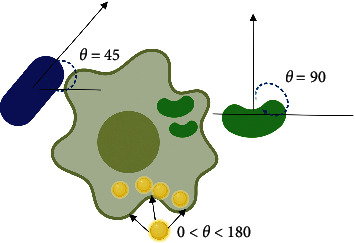
Effect of contact angle on the internalization efficacy. Nanoparticles having a prolate ellipsoid morphology (major axis 0.35–2 nm, minor axis 0.2–2 nm) had the slowest internalization rate and the highest attachment rate in comparison to spheroidal morphology (radius 0.26–1.8 nm) and oblate ellipsoidal nanoparticles (major axis 0.35–2.5 nm, minor axis 0.2–2 nm) [37].

When NPs come into contact with macrophages, the contact angle that initially occurs subsequently dictates the rate of internalization. A particle that aligns with its long axis parallel to the cell membrane would be internalized more slowly than NPs that align with the short axis parallel to the cell membrane. The rod-shaped NPs are internalized more quickly when they are perpendicular to the axis of the cell *θ* = 90°. When the NPs are tangential to the macrophage membrane, the rate of internalization decreases (Figure 4). The rate of spherical NP internalization is independent of *θ* due to their symmetrical shape [38, 39].

Thus, it can be concluded that the size, shape, and the aspect ratio are the major factors that affect the macrophage uptake of NPs.

### 3.3. Effect of Nanoparticle Charge on Biodistribution

The surface charge on NPs is usually measured as the zeta potential (*ξ*). Positively charged NPs (*ξ* > 10 mV) will induce serum protein aggregation, negatively charged NPs (*ξ* < −10 mV) exhibit strong reticuloendothelial system (RES) uptake, and neutral NPs (within ±10 mV) exhibit the least RES interaction and the longer circulation time [40].

Negatively charged NPs have a higher diffusion coefficient and penetrate the skin more rapidly, whereas positively charged NPs show the opposite behavior [27]. The potential charge effect could act as a repulsive or attractive force between the tissue surface and negatively or positively charged NPs, respectively. Therefore, Levchenko et al. concluded that neutrally charged NPs could be a better choice to eliminate the influence of surface charge [41].

It seems that the distribution of NPs in the kidney is not affected by the NP charge [42]. Positively charged NPs tend to accumulate more in the lungs than in other tissues. This is probably due to their ability to form aggregates by interacting with blood cells by electrostatic interactions and then these aggregates becoming entrapped in small capillaries in the lung [43].

Hepatic clearance can be influenced by the NP surface charge. NPs with high negative (<−10 mV) or positive (>10 mV) surface charge were efficiently cleared by the liver Kupffer cells from the blood circulation [44]. Another study found that NPs with *ξ* < −40 mV showed more than 90% clearance in 10 min compared to <10% clearance for the neutral NPs (*ξ* ±10 mV) and also increased liver uptake (60% ID versus <20% ID in 1 hour) [41]. In Table 2, we summarize the effect of NPs charge on tissue biodistribution.

### 3.4. Nanoparticles Surfactants and Biodistribution

NPs deployed in vivo can be protected from the immune system using various types of coating. Polyethylene glycol (PEG) has been used widely because it is biocompatible, chemically inert, and soluble in water and organic solvent [48]. PEG can also reduce phagocytic uptake, thereby prolonging the half-lives of NPs in a mechanism called the stealth effect [49]. PEGylated NPs generally accumulate in the liver at one-half to one-third of the amount compared to non-PEGylated NPs [50] and gave a significant reduction of accumulation in the spleen, liver, and pancreas [51]. Similarly, when coating NPs with other types of coating such as breviscapine (BVP), researchers found that the NPs were mainly distributed in the liver, spleen, heart, and brain [52].

Despite the advantages of using PEG as coating, several new reviews describe the immunogenic properties of PEG which is characterized by the production of antibodies against PEG after the first injection of PEG-NPs. This causes accelerated blood clearance (ABC) after the second injection [53–55]. One possible approach to reduce the immunological issues is to use immunosuppressive agents specific against PEG. For example, Tung et al. developed hybrid antibodies that can selectively deliver PEGylated medicine to the target cells [56]. Another strategy is the use of novel hydrophilic polymers other than PEG including BVP, polymeric NP, Poly (hydroxyethyl-L-asparagine), protein polymer, poly (amino acid) based stealth liposome [55], and zwitterionic polycarboxybetaine [57–60].

Many factors affect the PEGylation of NPs including the PEG polymer identity, the composition, density, hydrophobicity, and the nature of the proteins. These criteria should be properly regulated and adapted to avoid unfavorable effects of PEGylation [61]. NPs coated with lower molecular weight PEG were eliminated quickly from circulation [61].

In Table 3, we summarize the effect of using different types NPs coating on biodistribution.

## 4. Physicochemical Properties of NPs Affecting Tumor Penetration

The physicochemical properties of NPs (size, shape, charge, and surface coating) influence both tissue biodistribution and tumor uptake. Some properties play a major role on biodistribution and a minor role in tumor uptake. Furthermore, some properties are more pronounced in vitro than in vivo. In this part, we will discuss each property and how it may affect the tumor uptake.

### 4.1. Effect of Nanoparticles Size on Tumor Penetration

Intravenously administered NPs should be able to circulate in the bloodstream for a long time to have a good chance of reaching the tumor vasculature and then extravasating into the tumor tissue. Additionally, these NPs should not cross the vessel walls in normal tissues thereby causing adverse effects. As the pore size of normal vessels is between 6 and 12 nm, this would suggest that nanoparticles should be larger than that size [67]. The primary design requirement is that NPs should be able to pass through the pores of the leaky tumor vessels but not the pores of the normal vessels. The pores of the tumor vessels are generally between 40 and 200 nm [28].

The next consideration is the interaction between NPs and the openings in the tumor vessel wall. There are three kinds of interactions: hydrodynamic interactions due to forces induced by the motion of the particles within the fluid medium; steric interactions due to collisions of the particles with the wall; and electrostatic interactions due to attraction or repulsion between charged particles and the negatively charged glycocalyx on the surface of the vessel wall [68–70].

These types of interaction are controlled by the ratio between the sizes of the particles and the size of the openings in the vessel wall. When this ratio is small, transport is facilitated, whereas the transport of particles that approach the size of the openings is hindered and the particles are unable to pass through the wall [35]. Most of the studies confirm that the smaller the NPs size, the greater the likelihood of penetrating the tumor [71–74].

Different techniques can be used to alter the NP size. Wong et al. proposed a multistage system where 100 nm NPs (QDGelNPs) with a core composed of gelatin and a surface covered with quantum dots (QDs) “shrink” to 10 nm nanoparticles after extravasation from leaky regions of the tumor vasculature. Protease enzymes in the tumor degrade the 100 nm gelatin NPs, releasing smaller 10 nm NPs from their surface [75]. Tong et al. developed photo-switchable NPs to enhance penetration. By triggering the NPs (spiropyran and lipid: SP*-*C9 NPs) with UV light (365 nm, intensity = 1 mW/cm^2^), they underwent a reversible size change from 150 nm down to 40 nm. Consequently, the photo-switching could allow the fluorescence to increase and the release of drugs inside the cells [76].

By referring to Table 1 (study of the NPs size biodistribution) and Table 4 (NPs size effect on tumor uptake), it can be concluded that there is a strong need to develop nanomedicines with tailorable sizes and physicochemical properties to target the tumor microenvironment for effective delivery into deep tumors and limited extravasation in normal tissues for enhanced therapeutic efficiency. The variation of the size of NPs presents a bell-shaped curve with regard to tumor accumulation (extravasation within the tumor), where smaller sizes (below 80 nm) are not effective and larger sizes are also not suitable [78]. Similarly, the tumor penetration (movement within the tumor) of NPs also shows a bell-shaped curve, where the maximum NP penetration is less than 20 nm and may even be better for smaller sizes (between 2 and 15 nm) [70, 73, 80]. Hence a conflict might exist between these two concepts; the best tumor accumulation due to the EPR effect requires a NP size between 100 and 150 nm, while the best penetration requires a smaller NP size less than 12 nm. To address this discrepancy, different techniques have been recently tested including the programmable loaded NP [76] and the photo-switchable NPs discussed above [75].

### 4.2. How Important Is the Size in Determining the Tissue Penetration?

**Figure 5:**
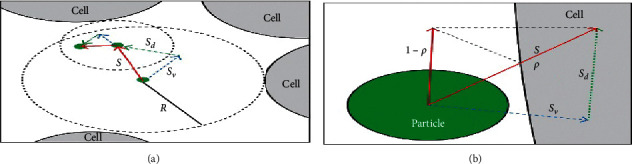
Illustration of the time-adaptive BD algorithm: the green circle represents the NPs, the large gray area represents the cells, and *P* is the probability of NPs to be captured by the NPs (adapted from [81]; open access no permission required).

The behavior of the NPs in contact with the cell membrane was studied in detail by Islam et al. in order to elucidate the effect of particle size that came into contact with the cells; they developed a model which enabled multiscale simulations under both diffusion and advection (horizontal flow) conditions (see Figure 5) [81]. They found a nonlinear relationship between tissue penetration and cellular uptake; this would suggest that cellular uptake is not determined by the probability of the NPs to be captured by the tumor surface, but by other factors including the likelihood of collisions and cell-particle interactions. They concluded that particle-size effect may dominate in a cell-free system in the absence of cell-particle interactions, and this effect is more pronounced in vitro. On the other hand, in vivo studies have shown that particle-cell interactions may moderate the particle-size effect, which might be the primary determinant of tissue penetration [81].

### 4.3. Effect of Nanoparticles Shape on Tissue Penetration

Nanoparticles can have different shapes, including filamentous, spherical, rod-like, or discoidal. Different techniques can be used to create a specific shape, including jet and flash imprint lithography (J-FIL) [50], film stretching, and nanofabrication processes [82, 83].

Filamentous nonmaterial (e.g., potato virus X) has been reported to have superior tumor-homing and pharmacokinetic properties compared to spherical particles [76]. Similarly, Champion et al. [84] compared different NP shapes; they concluded that elongated particles with a higher aspect ratio (i.e., the ratio of length to the width of NPs) are less prone to removal by the immune system [8]. In addition, elongated particles exhibited longer blood circulation times and avoided phagocytosis, depending on the angle of contact when they encounter macrophages [84].

Filamentous NPs (filomicelles) persisted in the circulation longer than spherical particles. When they are PEGylated (i.e., coated with polyethylene-glycol), this effect of filomicelles was further enhanced. Filomicelles have been shown to enter cells under static incubation conditions [84].

Table 5 summarizes how the shape of NPs may affect tumor penetration and accumulation.

#### 4.3.1. Effect of Shape on Vessel-Wall Margination

After escaping uptake by the macrophages, NPs should be able to marginate toward the wall of the blood vessels. Spherical NPs tend to remain in the center of the blood flow resulting in a decreased binding rate to the cells, whereas rod-shaped NPs tend to undergo a lateral drift depending on the orientation of their angles of contact [94, 95]. The tendency for rods to drift may be explained by the variable drag forces and torque forces that are exerted on the rods under flow conditions, which influences their ability to marginate. In fact, discoidal (AR = 0.5), hemisphere, and ellipsoidal particles (AR = 0.5) also have higher drift velocities than spheres. For different classes of discoidal particles, the drift velocity increases as the particle aspect ratio deviates further away from unity. Comparing ellipsoids, hemispheres, and discs, it was found that discoidal particles mostly followed highly oscillatory trajectories that led to increased interactions with the vessel wall [96].

#### 4.3.2. Effect of NPs Shape on Binding Capacity

Once NPs succeed in margination to the wall of the blood vessels, they have to be transported to their target site through a combination of binding and diffusion. NPs are able to accommodate many targeting ligands on their surface, unlike small-molecule drugs [97, 98]. It is very important to note that the binding capacity is influenced by many factors, not only the ligand-binding affinity.

The effect of the NP shape on tumor internalization is somewhat controversial. Some studies report the advantage of rod and cylindrical shaped NPs over other shapes, while, on the contrary, other studies have found better internalization for spherical shapes compared to nonspherical shapes [97, 98].

**Figure 6:**
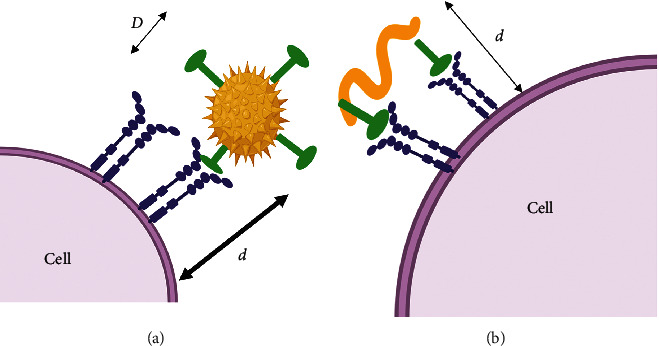
How different NP shapes could affect the binding avidity of NPs. *d* is the distance between the receptor and the NP and *D* is the diameter of the NP.

One important concept should be introduced when we are discussing the binding capacity of NPs, that is, the active fractional area (AFAC). For a sphere, the AFAC is defined as (*L* − *d*_B_)/*D*_c_(Figure 6), where *L* is the length of the ligand; *d*_b_ is the binding distance between the NP and the binding site; and *D*_c_ is the diameter of the NP [99–102]. Spherical NPs have different values of AFAC, whereas particles with equal surface areas have a similar binding capacity (Figure 6).

Gratton et al. [103] found that there was a difference in the internalization of two types of NPs having the same volume: rod-like (depth, *d* = 150 nm; height, *h* = 450 nm; volume = 0.00795 m^3^) and cylindrical counterparts (*d* = 200 nm, *h* = 200 nm, and volume = 0.00628 m^3^). Rod internalization occurred more rapidly and effectively than cylindrical shapes indicating that the shape had an important role in the internalization process [103]. In contrast, Agarwal et al. [85] found that low aspect ratio cylindrical NPs (*H*/*D*≈0.3; disk-like particles, 325 nm diameter and 100 nm height) showed a maximal intratissue delivery and a uniform degree of penetration compared to nanorods. They also found that decreasing the aspect ratio and size by increasing the diameter resulted in enhanced delivery efficiency, while for the rods their efficiency was similar, irrespective of their aspect ratio [85]. They suggested that when NPs actually reach the tumor, it is advantageous to have a very low aspect ratio. NP penetration is a balance between several factors: the surface area that allows the particle to interact either actively or passively with the tumor, the contact angle that predicts the rate of internalization.

There is an urgent need to design smart NPs that initially have a high aspect ratio (rod, filamentous, etc.) to achieve better circulation, then to change into a low aspect ratio (spherical shape) when they come into contact with the tumor to achieve better internalization.

### 4.4. Effect of NPs Charge on Tumor Penetration

Almost all of the components of the tumor microenvironment carry an electrostatic charge. The glycocalyx causes the blood vessels to be slightly negatively charged, whereas the hyaluronic acid existing in the interstitial space and the collagen fibers carry a small positive charge. Consequently, the electrostatic interaction between the tumor microenvironment and the NPs plays an important role in drug delivery [104].

A mathematical model was developed to determine how the NP surface charge affects the transport across the vessel wall [105]. When the pores of the tumor vessel wall are approximately 100 nm, the electrostatic repulsion is negligible, and the transvascular transport of negatively charged NPs is only hindered when the pore size is comparable to the Debye length (distance over which significant charge separation would occur) [105]. The range over which the electrostatic interaction is significant is determined by the Debye length. Electrostatic forces are strong when the Debye length is comparable to the diameter of the pores, because the particle and the electrostatic double layers of the pore are close to each other. An increase in the pore size causes the double layers to separate and only particles that are close to the wall of the cylindrical pore will interact [106].

A new concept relating to charge density has been introduced, in which the charge of the NP is a function of the pore size. Electrostatic attraction that improves transvascular flux is in competition with hydrodynamic and steric interactions [90]; with smaller pores sizes (<100 nm), hydrodynamic and steric forces dominate, and, as a result, the electrostatic interaction is negligible. When the pore size increases, the electrostatic interaction becomes more dominant. [107]; finally, if the pore size is too large (>300 nm) compared to the NP size, all three types of interaction decrease, and the electrostatic interaction entirely disappears [106].

In order to deliver the appropriate NP to a specific cancer site, a deep understanding of the type of tumor is crucial. For example, in breast cancer, the pore size exceeds 1 *μ*m in diameter, while pancreatic and brain tumors have relatively small pore sizes. In addition, a value of surface charge density exists for every NP preparation [107].

Surface charge is another key parameter that determines the NP performance, due to the fact that tumor cells are slightly negatively charged. Therefore, it is considered that positively charged NPs could be taken up better by the cells due to “electrostatic adhesion-mediated targeting” [108, 109].

Neutral or negatively charged NPs may travel for longer distances inside the tumor tissues than positively-charged NPs. Thus a “delayed charge reversal profile” could be a good choice because the tumor penetration could be enhanced without affecting the cellular internalization [42, 110]. Gou et al. [111] found better intratumoral penetration and stronger tumor growth inhibition when preparing NDDS with delayed charge reversal by decorating NP with PGlu-g-mPEG at low PH. They monitored the charge by the *E* potential [111].

Positively charged NPs have been shown to better target tumor vessels, but after extravasation, a switch to a neutral charge allowed more rapid diffusion of the NPs within the tumor tissue [105]. In Table 6, we summarize the effect of NPs charge on tumor uptake.

By referring to Tables 2 and 6, the optimal charge for NPs should initially be neutral for better biodistribution and then switching into cationic or slight negative for selective tumor targeting, moreover, neutral NPs could perform better after entering the tumor tissue.

### 4.5. NP Surface Coatings and Tumor Penetration

Coating NP is elementary to achieve better circulation time and reduced phagocytosis, whereas, in contact with the tumor tissue, many studies found that PEG may act as an obstacle hindering the interaction of the NPs with the target cells. Some proteins are capable of translocating through the cell membrane efficiently without compromising their integrity [72, 114, 115]. These molecules include cell penetrating peptide (CPP), avidin-biotin, saccharides, and transferrins [116–118]. Tan et al. found that CPP mainly affects transport and exocytosis, whereas PEG polymer influences mucus penetration [119]. Approaches to overcome this limitation while still maintaining the advantages of PEG have been tested, such as preparing PEG covalently linked to 1,2-dioleoyl-sn-glycero-3-phosphoethanolamine (DOPE). At low pH (5-6), the conjugation linkage will be hydrolyzed, leading to fusion with the endosomal membrane and releasing the contents into the cytosol [120].

In a similar approach, Kale and Torchilin [121] prepared TAT (cell penetrating peptide) liposomes with a cleavable PEG coating. When the pH was reduced to 5 or 6, the PEG chains will be released, so the TAT will be better internalized by the cancer cells [121].

This promising strategy to improve the penetration capacity of NPs is hampered by the lack of cell specificity and the mode of delivery is not well understood. In an attempt to inhibit the nonspecific interaction of CPP with the blood stream, a novel strategy has been introduced by Ding et al. [118] using ligant-switchable NPS with a hidden CPPs under the PEG corona to avoid direct interaction of immune cells and normal tissue, once they extravasate from the blood vessels the acidic tumor environment would trigger the CPP exposure enabling the diffusion into the tumor [118].

Liu et al. studied the effect of surface charge and the particle size of CPP on cellular internalization. They found that the zeta potential is the key predictor of transduction efficiency, whereas the size of the CPP has a minor effect on cell permeability [122]. More investigations are required to improve their tumor delivery and to reduce their possible side effect [123]. A combination of unconventional electron microscopy technique helps in determining the molecular mass distribution and compositions of dendrimers NPs which helps in assessing the composition, mass, and homogeneity of metal containing organic NPs [124].

In Table 7, we summarize the effect of different NPs coating on tumor penetration.

## 5. Discussion

The major factors that improve the tissue biodistribution of NPs are the physicochemical properties, whereas the major factor that affects the cellular uptake is the coating.

From Tables 1 and 2, it can be concluded that larger NPs (larger surface area) escape the macrophage and RES uptake and circulate for longer times, whereas smaller-sized NPs (<12 nm) have better tumor uptake. A possible explanation is that although NPs with large surface area can more likely come in contact with the cells, which increase the likelihood of binding, they may not be easily endocytosed by nonspecific cell uptake pathways. Instead, large NPs must be taken up into the cells via an active pathway, which requires the cells to expend more energy to accomplish the task. On the other hand, small NPs do not have much available contact surface area with cells, so adhesion is typically not strong; however, the smaller NPs can be absorbed and taken up by the cells much easier.

In terms of which shape would be best, it can be concluded from Table 3 that to achieve better tissue biodistribution and decrease macrophage uptake, a higher aspect ratio would perform best (particularly for rod and filamentous shapes) compared to other forms. However, for better tumor uptake, it is likely that the best shape depends on the type of cells involved. Tumor uptake of NPs is a dynamic process, which varies as the stage of the tumor progresses. Uhl et al. [93] concluded that the best shape for transporting drugs across the EC barrier shifted from the larger long rod-shaped NPs to smaller NPs. This occurs because the EC monolayer regains confluency as the tumor regresses, which in turn impedes the transport of the larger NPs [93].

Ideally, a combination of the three different shapes deployed at different times could be the best for getting drugs to the tumor. Specifically, starting with large NPs, which can transport high amounts of the drug early in the treatment time frame, followed by small NPs as the vasculature recovers, and the transport of large bulky NPs becomes increasingly difficult [93].

The key factor that determines the efficacy of passive targeting is the surface area and size of the NPs. Nanoparticles of different sizes behave in a qualitatively different manner. Dqivedi et al. [114] concluded that despite their efforts to enhance hydrophobicity and testing different surface charges, there are cases where larger NPs are totally excluded from cells without any significant NPs uptake [114]. As NPs decrease in size, the physicochemical properties such as surface charge and hydrophobicity become more pronounced.

It seems that the so-called EPR effect is very size-dependent, and it can be slow and not very efficient compared to active targeting [115]. In addition, EPR is also likely to operate in some nontumor vascular beds; it is effective only on well-vascularized primary tumors and ineffective in metastatic disease especially with small metastatic deposits [115, 126].

Furthermore, using a mathematical model, Islam et al. [81] found that the NP-cell interactions may moderate the particle size effect. The addition of coatings and surfactants such as peptides (Tat, PEG, and BVB) becomes important in order to achieve an active targeting of NPs that does not rely solely on the EPR effect. Moreover, much effort has gone into the design of molecular ligands such as vitamins, hormones, and growth factors that can be attached to the outside of NPs and which are specifically recognized by receptors on the cancer cells.

A very important point should be noted here is that the addition of coatings and ligands to NPs does not, in itself, have much effect on tissue distribution, which is mainly dependent on the physicochemical properties of the NPs. The ligands and coatings act to improve the intracellular uptake by the target cells and do not necessarily have any effect in improving the tumor targeting [120, 121, 127]. Some studies nowadays discovered the immunogenic properties of PEG which is manifested by the production of anti-PEG antibodies after the first injection of PEG-NPs causing a rapid clearance and ABC phenomenon in the second injection. Novel hydrophilic polymers other than PEG may be a potential alternative choice [53–55, 57, 58].

Once the NPs succeed in coming in close contact with the target cells, it is more advantageous to remove the surfactant coating to achieve better cellular uptake as it has been shown in many studies that the ligands may be an obstacle for effective cellular uptake [128, 129].

**Figure 7:**
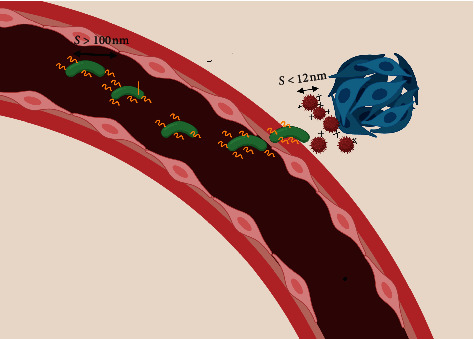
Proposed ideal NPs characteristics. When NPs are in the blood circulation, it is advantageous to have a larger size (>100 nm) nonspherical shaped NP, with a neutral charge to achieve better circulation and tumor accumulation. Once in contact with the tumor, it is more advantageous to have positively charged or slight negatively charged NPs with a smaller size (<12 nm) and a lower aspect ratio. The surfactants should be removed once the NPs enter the extracellular matrix (ECM).

In Figure 7, we summarize the ideal NP behavior. Ideal NPs should change their physicochemical properties once they have come into close contact with the target cells. These changes could include their size, shape, charge, and coatings. Using an external energy source (laser or heat) [130–132] or taking advantage of internal stimuli (pH or redox) [133–135] or a tumor feature such as a particular enzyme activity could all be tested to achieve maximum tumor targeting efficacy.

## 6. Conclusions

Smart or stimulus-responsive NPs could allow them to act as a type of “nanorobot,” having certain properties while circulating in the blood circulation and changing their properties when they come into contact with the tumor. These changes could include not only the size, but also the shape, the charge, and the coatings.

It seems nowadays that the concept of “one fits all” does not apply anymore. Nanocarriers should be customized to the specific target to achieve the best result. Furthermore, local cancer therapy such as subcutaneous administration starts to be the preferred administration root, since smaller volumes can be injected, meaning lower injection times and shorter hospitalization for patients [136].

Important questions that require answers include what technology we need to safely and precisely manipulate the nanoparticles properties and what is the influence of the administration route on the tumor biodistribution and tumor uptake. Developing effective strategies to modify tumor properties such as degradation of the extracellular matrix is another field to be investigated in the near future.

## Figures and Tables

**Table 1:** Biodistribution of NPs with different sizes in the liver, spleen, tumor, kidney, brain, and lung.

Authors	NP size	Liver	Spleen	Tumor	Kidney	Brain	Lung	Other
Li et al. [32]	6.2, 24.3, 42.5, and 61.2 nm mean diameter	High 42.5 and 61.2 nm	High 42.5 and 61.2 nm	6.2 and 24.3 nm	6.2 and 24.3 nm	6.2 and 24.3 nm	6.2 and 24.3 nm	
Sonavane et al. [27]	15, 20, and 100 nm	High % for all	High % for all	15 > 20>100 nm	High % for all	(i) 15 nm low % (ii) 20 nm absent (iii) 100 nm absent	(i) 15 and 20 nm high % (ii) 100 nm low %	(i) 15 nm absent (ii) 20 and 100 nm traces
Tate et al. [33]	20 and 100 nm	High 20 nm < 100 nm	High for all	20 > 100 nm	20 < 100 nm	Traces	High	—
Takeuchi et al. [34]	20, 50, and 100 nm	High % for all	High for all	20 > 50>100 nm	20, 50, and 100 nm Moderate	(i) 20 nm moderate% (ii) 50 and 100 nm absent	Moderate	Traces
Dziendzikowska et al. [35]	−20 and 200 nm silver NP(AgNPs)	(i) 20 nm high % (24 h) (ii) 200 nm moderate %	(i) 20 nm high (7 days) (ii) 200 nm low	Absent for both	(i) High % 20 nm (ii) low %200 nm	(i) 20 nm Moderate % (ii) 200 nm low %	(i) 20 nm high % after 7 days (ii) 200 nm low %	(i) 20 nm traces (ii) 200 nm absent

It can be concluded that the smaller the size of NPs, the more accumulation found in the spleen, liver, and lung than the kidney; a moderate concentration could accumulate in the tumors and only a low quantity is able to cross the blood brain barrier to accumulate in the brain, whereas the higher the size (+100 nm), the greater distribution in the liver, kidney, and spleen. None of these studies found a good distribution in the brain and only traces of large diameters were found in the tumor.

**Table 2:** Effect of different NPs charges on tissue biodistribution: charged NPs are cleared rapidly by the immune system.

Authors	NPs type	Charge	Uptake	Biodistribution
Xiao et al. [44]	PEG-micellar nanoparticles	Surface charge: high negative (<−10 mV) and high positive	High uptake by slightly negative charged NPs	All charged NPs cleared by the Kupffer cells
He et al. [45]	Polymeric NPs	Negative (−10 mV) neutral and positive charge (+35 mV)	High uptake of slight negative charged NP	Liver > spleen > lung > tumor > kidney > blood
Walczak et al. [46]	Polystyrene NPs 50 nm	Negative (−7, 7 mV) positive NPs and neutral	High uptake of negatively charged NPs than the neutral and positive one	Kidney > heart > stomach > small intestine
Verma et al. [47]	Encapsulated paclitaxel	Negative pectin NPs	—	(i) Major accumulation in liver > kidney > lung > spleen (ii) Tissue prolonged plasma retention

**Table 3:** Effect of NPs coating on tissue biodistribution.

Authors	Nanoparticle	Coating added	Distribution
Zhang et al. [60]	Gold NPs	Zwitterionic polycarboxybetaine (PCB)	PK behavior was unchanged, no antiuricase detected, no anti-PCB antibodies detected
Rodriguez et al. [62]	160 nm nanobeads	CD47 “self” peptides	Prolonged drug circulation by delaying phagocytic clearance by the liver and spleen
Kreuter et al. [63]	poly(butyl cyanoacrylate) nanoparticles	Polysorbate 80	enhanced drug delivery beyond the blood-brain barrier
Parodi et al. [64]	Nanoporous silicon particles (NPS)	Membranes purified from white blood	Prolonged circulation time
Hu et al. [65]	Polymeric nanoparticles	Erythrocyte membrane	Prolonged circulation time
Romberg et al. [55]	Liposome	Poly(hydroxyethyl-L-asparagine) (PHEA)	Longer blood circulation times than PEG liposomes. The second injection less rapidly cleared from the circulation than the second dose of PEG liposomes
Lila et al. [66]	Liposome	Polyglycerol (PG)	Reduced effect of ABC when using polyglycerol compared to PEG

**Table 4:** Effect of NPs size on tumor penetration: the smaller the size, the higher the probability of tumor uptake.

Author	Nanoparticle type	Nanoparticle size	Tumor type	Tumor penetration efficacy
Cabral et al. [71]	Drug loaded polymeric micelles	30, 50, 70, 100 nm	Two cancer type (high and low permeable)	Only 30 nm penetrate poorly permeable cancer
Ezealisiji and Okorie [72]	Silver NPs	22, 58, 76, 378 nm	Dermatological application	22 nm exhibit the highest cumulative amount (penetration)
Arvizo et al. [77]	Gold NPs (without any surface modification)	5, 10, 20 nm	Human umbilical vein endothelial cells	20 nm Maximum effect anti-angiogenic effect(VFGF inhibition)
Peretz et al. [78]	Gold nanoparticles	15, 30, 90, 150 nm	Head and neck cancer cells	15 nm best binding capacity to cancer cells & 90 nm is optimal for cell targeting and tumor accumulation
Popović et al. [73]	Quantum dots	12, 60, 125 nm	Melanoma in mouse	Rapid penetration for `12 nm NP
Sonavane et al. [27]	Gold nanoparticles	15, 50, 100, 200 nm	Mice (different organ), intravenous administration	15 nm wide organ distribution, only 15 and 50 nm pass blood brain barrier
Huang et al. [79]	PVP-coated iron oxide nanoparticles (PVP-IOs)	37–120 nm	Hepatic lesion in mouse	37 nm greatest cellular uptake
Hemant et al. [28]	Gold NPs	1 to 125 nm (intravenous)	Different pore size	Rapid penetration for `12 nm NP
Huang et al. [80]	Gold nanoparticles (AuNPs)	2, 6, 15 nm	Breast cancer cells	2 and 6 nm Maximum tumor uptake and permeability. 2 & 6 nm found in nucleus and cytoplasm whereas 15 nm only in cytoplasm

**Table 5:** Effect of different shapes of NPs on tumor penetration.

Author	Nanoparticle type	Nanoparticle shape used	Type of treatment	Efficacy
Agarwal et al. [85]	Gold NPs	Nanohydrogel; cylindrical; nanorods; spherical NPs	3D spheroid model	Better effect of cylindrical hydrogel NPs
Christian et al. [86]	Micelles	Filamentous and spherical	Mouse xenograft tumor	High tumor accumulation of filamentous
Lui et al. [87]	Single-walled carbon nanotube (SWNT)	Carbon nanotubes	Cancer in mice	Tumor targeting effect
Bartczak et al. [88]	Gold NPs	Spherical, rod, hollow, silica-gold, core shell	Human endothelial cell uptake	High cellular uptake for the spherical and the lowest for the hollow shapes
Tak et al. [89]	Shaped silver NPs (AgNPs)	Rods, spherical, triangular	Skin permeability in hairless mice	Nanorods had maximum penetration
Champion et al. [84]	Nonspherical polystyrene particles	Spherical and filamentous	Cancer	Spherical shapes showed better tumor homing
Champion and Mitragotri [84]	Non-cross-linked polystyrene (PS)	Spheres, ellipsoids, elliptical disks, prolate ellipsoids, rectangular disks	Uptake by macrophages (phagocytosis)	Elongated NPs showed negligible phagocytosis
Kessentini and Barchiesi [90]	Gold NPs	Nanorods; spheroids; cylinders; capped cylinders, nanoshells; hollow nanospheres	Shallow skin cancer and deeper cancer	(i) Nanospheres for shallow cancer (ii) Nanospheres and nanorods for deep cancer
Bruckman et al. [91]	PEGylated tobacco mosaic virus	Nanorods and nanospheres	Blood circulation	Prolonged circulation of nanorods better than nanospheres
Geng et al. [92]	Paclitaxel-loaded filomicelles	Spherical and filamentous (filomicelle)	Blood vessels of rats and mice	Longer circulation of filomicelles
Uhl et al. [93]	Polymeric NPs	Sphere, short rod, long rod	Microfluidic device (transport in blood vessel)	Best treatment required combination of different shaped NPs administered at different times

All the studies agreed that nanorods, discoidal, and micelles showed better tumor targeting accumulation and longer circulation. One study found better cellular uptake of spherical NPs compared to other shapes.

**Table 6:** Effect of NPs surface charge on tumor uptake: it seems that positively charged NPs and slightly negative display a good tumor uptake.

Authors	Nanoparticle types and charge	Treatment/study	Efficacy
Xiao et al. [44]	NPs with high negative and high positive charge	Tumor cellular uptake	High uptake of slight negative and slight positive NPs
Gou et al. [111]	NPs with different charge	Tumor cell uptake	Delayed charge reversal strategy could improve therapeutic effect
Graf et al. [112]	High positive NPs	Stability in physiological media	Effective cellular internalization
He et al. [45]	Polymeric NPs negative(−40 mV) and positive charge (+35 mV)	Tumor uptake	Slight negative charge accumulate more efficiently in tumor
Chen et al. [113]	Positive and negative NPs	Tumor uptake	High uptake of positively charged Nps increase uptake of both charge under hypoxic conditions
Stylianopoulos et al. [105]	Positive NPs switched into neutral inside the tumor	Cancer (tumor targeting)	Positive NPs: Effective tumor targeting & neutral charge allowed quicker diffusion of the nanoparticles to the tumor tissue

**Table 7:** Effect of adding different types of coatings to the NPs.

Author	NPs type and size	Type of added ligant	Treatment	Effect/distribution	Benefit
Liu et al. [52]	Coated BVP-PLA-NPs 177 and 319 nm	BVP	IV administration	Liver, spleen, heart, brain	NPs penetrate the BBB, avoid the RES, prolong the half-life of BVP
Takeuchi et al. [51]	Gold NPs 20, 40 and 80 nm	PEG	IV administration	Usefulness of PEG with smaller NPs size	Reduced accumulation in liver, spleen, improved delivery to the brain
Ezealisiji and Okorie [72]	Ag NPs	Peg, PG, Tween, NaLSo4	Dermatological application (skin penetration)	Maximum penetration efficacy of NaLSo4 followed by PG	Improved skin penetration
Hu et al. [125]	PEG-PLA nanoparticles	Peptide F3 PEG-PLA nanoparticles	IV treatment of glioma	Enhanced accumulation at the tumor site and deep penetration into the glioma parenchyma	Improved parenchyma penetration

The maximum penetration was achieved by the NaLSO4 followed by PG and then PEG [105]. Coated BVP-PLA NPs also avoided the RES and prolonged the half-life of NPs [111].

## Abbreviations

ABC: Accelerated blood clearance
BVP: Breviscapine BVP
CPP: Cell penetrating peptide
ECM: Extracellular matrix
EPR: Enhanced permeability and retention
FDA: Food and Drug Administration
J-FIL: Jet and flash imprint lithography
MPS: Mononuclear phagocytic system
NP: Nanoparticle
PCC: Personalized protein corona
PEG: Polyethylene glycol
PLA: Polylactic acid
QDs: Quantum dots
RES: Reticuloendothelial system
UV: Ultraviolet.
